# AKR1B10 promotes breast cancer cell migration and invasion via activation of ERK signaling

**DOI:** 10.18632/oncotarget.16624

**Published:** 2017-03-28

**Authors:** Jia Li, Yuanwei Guo, Lili Duan, Xinglin Hu, Xi Zhang, Jian Hu, Li Huang, Rongzhang He, Zheng Hu, Weihao Luo, Tan Tan, Renbin Huang, Duanfang Liao, Yuan-Shan Zhu, Di-Xian Luo

**Affiliations:** ^1^ Translational Medicine Institute, National & Local Joint Engineering Laboratory for High-throughput Molecular Diagnosis Technology, Affiliated to The First People's Hospital of Chenzhou, University of South China, Chenzhou 423000, P.R. China; ^2^ Center for Clinical Pathology, Affiliated to The First People's Hospital of Chenzhou 423000, P.R. China; ^3^ Department of Neurology, Affiliated to The First People's Hospital of Chenzhou 423000, P.R. China; ^4^ School of Pharmacy, Hunan University of Chinese Medicine, Changsha 410208, P.R. China; ^5^ Department of Clinical Pharmacology, Xiangya Hospital and Institute of Clinical Pharmacology, Central South University and Hunan Key Laboratory of Pharmacogenetics, Changsha, Hunan 410078, P.R. China

**Keywords:** breast cancer, AKR1B10, ERK

## Abstract

**Background:**

Aldo-keto reductase family 1, member B10 (AKR1B10), is known to be significantly induced in the cells of various cancers such as breast cancer. However, the mechanisms of AKR1B10 promoting tumorigenesis in breast cancer remain unclear. In the present study, we demonstrated the potential role and mechanism of AKR1B10 in the invasion and migration of breast cancer cells.

**Methods:**

The expression level of AKR1B10 in breast carcinoma, para-carcinoma and cancer tissues were detected by immunohistochemical evaluation and real-time polymerase chain reaction (RT-PCR), and the correlationships between AKR1B10 expression and clinicopathological features in breast cancer patients (n=131) were investigated. AKR1B10 was ectopically expressed in MCF-7 cells or silenced in BT-20 cells. The roles of AKR1B10 expression in the migration and invasion of MCF-7 cells and BT-20 cells were explored by wound healing assay, transwell migration assay and transwell matrigel invasion assay, and finally the activation level of extracellular signal-regulated kinase 1/2 (EKR1/2) activation and the expression level of matrix metalloproteinase-2 (MMP2) and vimentin in MCF-7 and BT-20 cells were measured by western blot.

**Results:**

We found that AKR1B10 expression was increased in malignant tissues, which was correlated positively with tumor size, lymph node metastasis (*p*<0.05). MCF-7/AKR1B10 cells displayed a higher ability of migration (43.57±1.04%) compared with MCF-7/vector cells (29.12±1.34%) in wound healing assay, and the migrated cell number of MCF-7/AKR1B10 was more (418.43±9.62) than that of MCF-7/vector (222.43±17.75) in transwell migration assay without matrigel. We furtherly confirmed MCF-7/AKR1B10 cells invaded faster compared with MCF-7/vector cells by transwell matrigel invasion assay. Finally, we found AKR1B10 induced the migration and invasion of MCF-7 and BT-20 cells by activating EKR signaling, which promoted the expressions of MMP2 and vimentin. PD98059, a specific inhibitor of the activation of MEK, blocked the migration and invasion by inhibiting the expression of MMP2 and vimentin.

**Conclusions:**

AKR1B10 is overexpressed in breast cancer, and promotes the migration and invasion of MCF-7 and BT-20 cells by activating ERK signaling pathway.

## INTRODUCTION

Breast cancer is the most common malignant tumor in female, and its incidence and mortality increased rapidly in recent decades. The long-term survival of breast cancer after surgical or chemotherapy treatment still remains poor, for its high recurrence and metastasis rates. Breast cancer metastasis is the main cause leading to the patients’ death [1].

Aldo-keto reductase family 1, member B10 (AKR1B10), is primarily expressed in human colon and small intestine but overexpressed in breast cancer, hepatocellular carcinoma, non-small cell lung carcinoma, cervical and endometrial cancers [2–9]. Our previous studies have shown that AKR1B10 is secreted through a lysosome-mediated non-classical pathway, which leads to the increase of AKR1B10 in the serum of breast cancer patients. And its expression level is positive correlated with the lymph node metastasis of breast cancer [2, 10]. However, the molecular mechanisms of AKR1B10 promoting the metastasis of breast cancer remain largely unknown. Thus, the exact mechanisms have been always required for exploring the use of AKR1B10 as therapeutic target in cancer clinics.

The extracellular signal-regulated kinase (ERK) signaling pathway is a major determinant in the control of diverse cellular processes such as proliferation, survival, differentiation and motility and is often activated in human tumors. This pathway stimulates the expressions of the matrix metalloproteinase (MMP) gene, which is required for the induction of cell motility via the degradation of the extracellular matrix, and vimentin gene, which is an important protein in deformation and movement of tumor cells. Elevated expression of MMPs and vimentin are associated with increased metastatic potential in many tumor cells [11].

In this study, we investigated the potential involvement of ERK signaling pathway in AKR1B10-associated breast cancer cell migration and invasion *in vitro*.

## RESULTS

### AKR1B10 is overexpressed in breast cancer tissues

**Figure 1:**
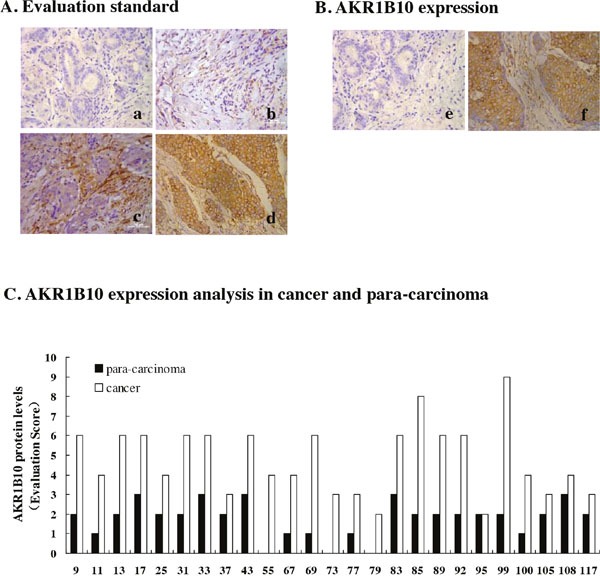
AKR1B10 expression in breast cancer tissues AKR1B10 protein expression was detected in 131 cases of breast cancer specimens by immunochemistry. **(A)** Immunohistochemistry staining evaluation standard of AKR1B10 expression levels were made by scoring on the staining intensities from 0 to 3+ : 0 for no staining (a), 1+ for weak immunoreactivity (b), 2+ for medium immunoreactivity (c), 3+ for strong immunoreactivity (d). **(B)** AKR1B10 expression was evaluated in breast para-carcinoma tissues and carcinoma tissue: e) para-carcinoma; f) breast carcinoma. **(C)** AKR1B10 protein levels were analyzed in breast cancer and para-carcinoma of randomly selected 25 cases and compared in para-carcinoma and breast cancer.

Protein levels of AKR1B10 was examined in 131 breast cancer specimens by immunochemistry. Expression levels of AKR1B10 were scored on the staining intensities from 0 to 3+ (Figure 1A), and its expression was detected in breast cancer tissue and para-cancerous tissue (Figure 1B). AKR1B10 protein expression levels of AKR1B10 (evaluation scores) in the cancer tissue were higher than that in para-carcinoma tissue in 24/25 cases (Figure 1C). The percentage of AKR1B10-positive specimens was 90.07% (118/131) in the breast cancer tissue (Table 1).

**Table 1:** Correlation of AKR1B10 expression with clinicopathological parameters (n=131)

Clinicopathological parameter	AKR1B10 expression level				Subtotal	X values	*p* values
	3	2	1	0			
**Subtotal**	58	33	27	13	131		
Age (years)						1.520	0.689
≤50	32	17	17	9	75		
>50	26	16	10	4	56		
Tumor size(cm)						10.767	0.012
≤4	30	14	22	6	72		
>4	28	19	5	7	59		
TNM stage						3.590	0.318
I- II	24	9	12	7	52		
III- IV	34	24	15	6	79		
Lymphatic metastasis						10.741	0.012
YES	44	15	13	8	80		
NO	14	18	14	5	51		
Distant metastasis						4.204	0.190
YES	2	3	4	0	9		
NO	56	30	23	13	122		

### Correlation of AKR1B10 expression with clinicopathological parameters

Among the patients of breast cancer, there were 56 cases aged >50 and 75 cases aged < 50; 72 cases with a tumor size <4cm and 59 cases with a tumor size > 4cm; 80 cases with lymph node metastasis and 51 cases without lymph node metastasis; 122 cases without distant metastasis and 9 cases with distant metastasis. The numbers of patients classified by TNM stage were as follows: TNM I/II(n=52), TNM III/IV(n=79). Through statistically analyzing correlationships between AKR1B10 expression and clinicopathological parameters, we found the expression levels of AKR1B10 were highly positively correlated with tumor size and lymph node metastasis.

### AKR1B10 promoted migration and invasion of MCF-7 cells and BT-20 cells

**Figure 2:**
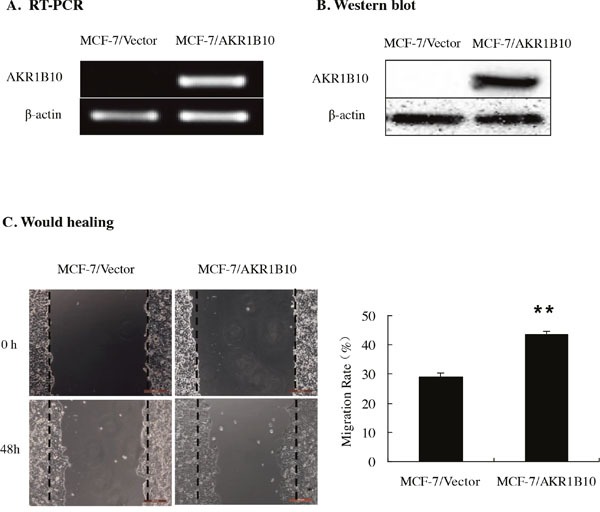
Effect of AKR1B10 on wound healing ability of MCF-7 cells **(A and B)** The expression levels of AKR1B10 were detected in MCF-7 cells by RT-PCR **(A)** and Western blot. AKR1B10 was ectopically expressed in MCF-7 cells. **(C)** The effect of AKR1B10 on the migration of MCF-7 cells by wound healing assay. Scale bar = 200μm. Representative images are shown on the left panel, and the statistical graphs on the right panel indicating the average number of cells per field 48 h after transfection (**p < 0.01, compared with MCF-7/Vector).

To evaluate the effects of AKR1B10 on migratory and invasive properties of MCF-7 cells, AKR1B10 was ectopically expression in MCF-7 cells. Then monoclonal MCF-7 cells with stable AKR1B10 expression was picked up. The mRNA and protein expressions of AKR1B10 in MCF-7 cells were measured by RT-PCR and Western blot (Figure 2A and 2B).

**Figure 3:**
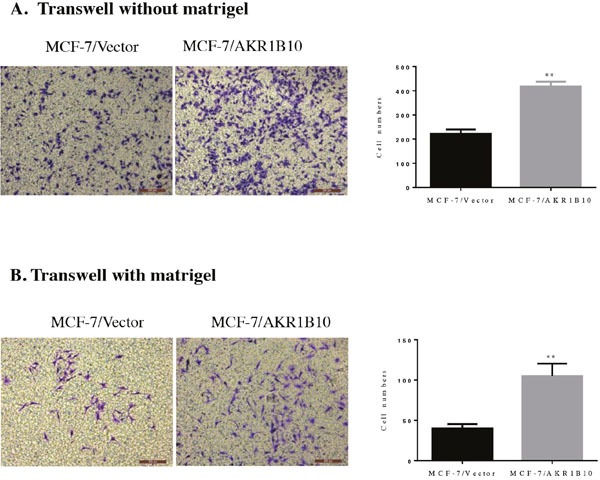
Effects of AKR1B10 on transwell migration and invasion abilities of MCF-7 cells **(A)** The effect of AKR1B10 on migration of MCF-7 cells was investigated by transwell migration assay without matrigel. **(B)** The effect of AKR1B10 on invasion of MCF-7 cells was investigated by transwell invasion assays with matrigel. Cell were counted after staining with 0.1% crystal violet. Scale bar = 200μm. Representative images are shown on the left panel, and the statistical graphs on the right panel indicating the average number of cells per field 48 h after transfection (**p < 0.01, compared with MCF-7/Vector).

Then we investigated the potential role of AKR1B10 in modulating the migration and invasion abilities of MCF-7 cells by wound healing assay, transwell migration assay and transwell matrigel invasion assay. Wound healing assays indicated that the migration rate of MCF-7 cells with AKR1B10 expression was 43.57±1.04%, higher than 29.12±1.34% of MCF-7 cells with vector, which suggested that AKR1B10 promoted the migration of MCF-7 cells (Figure 2C). To further identify these results, we performed transwell migration assays without matrigel and found that MCF-7/AKR1B10 cells displayed a higher migration ability (418.43±9.62) compared with MCF-7/vector cells (222.43±17.75) (Figure 3A). Next, transwell invasion assays with matrigel revealed that the invasion cell numbers of MCF-7/AKR1B10 was 105±15.47, faster than 40.27±5.47 of MCF-7/vector cells (Figure 3B). The effect of reduced expression of AKR1B10 on cell migration and invasion of BT-20 cell showed the same results (Figure 5D, 5E).

### AKR1B10 stimulates the migration and invasion of MCF-7 and BT-20 cells by activating ERK signaling

**Figure 4:**
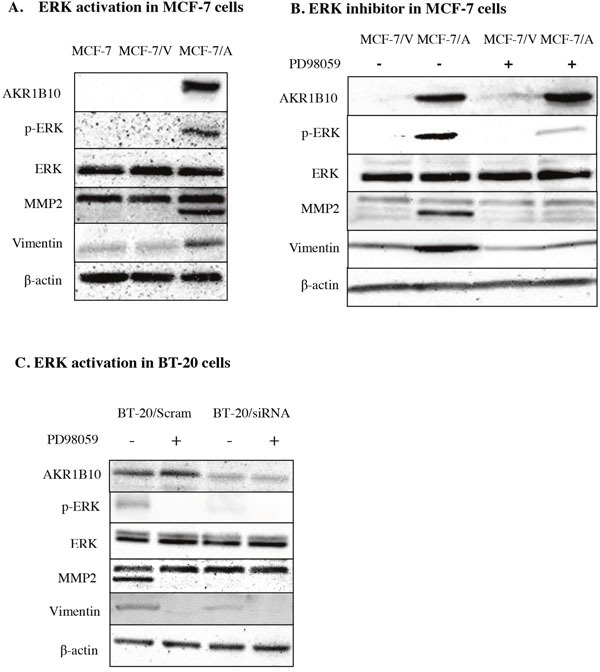
AKR1B10 promotes ERK signaling in MCF-7 cells and BT-20 cells **(A)** The expression levels of ERK, p-ERK, MMP2 and Vimentin were analyzed in MCF-7/AKR1B10 cells. (**B** and **C**) The AKR1B10-induced levels of p-ERK, MMP2 and vimentin were interrupted by ERK inhibitor PD98059 (10μM). Scram: Scramble siRNA; siRNA: AKR1B10 siRNA; MCF-7/V: MCF-7/Vector; MCF-7/A: MCF-7/AKR1B10.

**Figure 5:**
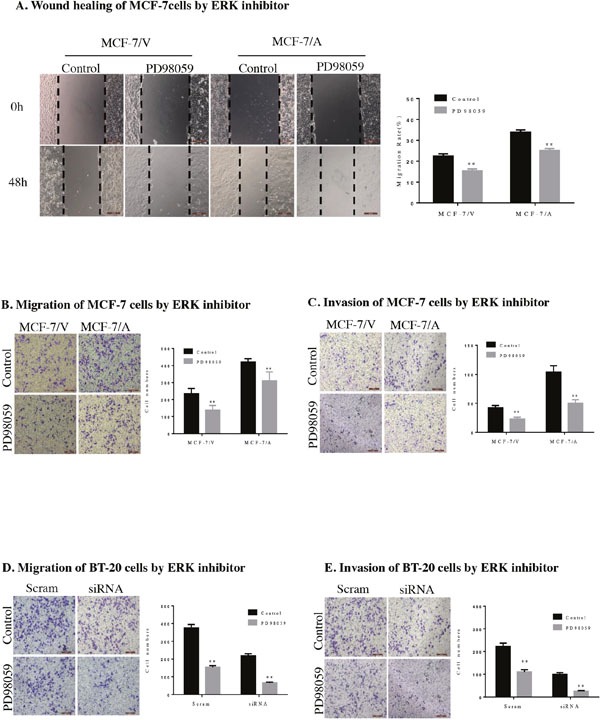
AKR1B10 promotes the migration and invasion of MCF-7 cells by ERK signaling pathway **(A)** AKR1B10-induced migration of MCF-7/AKR1B10 cells was inhibited by ERK inhibitor PD98059 by wound healing assay. **(B)** AKR1B10-induced migration of MCF-7/AKR1B10 cells was inhibited by ERK inhibitor PD98059 by transwell migration assay without matrigel. **(C)** AKR1B10-induced invasion of MCF-7/AKR1B10 cells was inhibited by ERK inhibitor PD98059 by transwell invasion assay with matrigel. (**p < 0.01, compared to MCF-7 cells with AKR1B10 expression or with a vector control). **(D)** Migration of BT-20 cells was inhibited by ERK inhibitor PD98059 by transwell migration assay without matrigel. **(E)** Invasion of BT-20 cells was inhibited by ERK inhibitor PD98059 by transwell invasion assay with matrigel (**p < 0.01, compared to BT-20 cells with AKR1B10 expression or with AKR1B10-siRNA control). Cell were counted after staining with 0.1% crystal violet. Scale bar = 200μm. Representative images are shown on the left panel, and the statistical graphs on the right panel indicating the average number of cells per field.

Then we investigated the potential mechanism of AKR1B10-mediating the migration and invasion of MCF-7 cells and BT-20 cells. We detected the expression level of ERK, p-ERK, MMP2, vimentin in MCF-7 cells and BT-20 cells by Western blot. The result showed AKR1B10 stimulated activation of EKR and expression of MMP2 and vimentin. Furthermore, we found the protein levels of MMP2, an enzyme regulated by EKR1/2 signaling pathway, was up-regulated in MCF-7/AKR1B10 cells and BT-20 cells (Figure 4A, 4C). Pharmacological inhibition of ERK signaling by MEK1/2 inhibitor PD98059 (10μM) attenuated the activation of ERK and the expression of MMP2 and vimentin in MCF-7/AKR1B10 cells and BT-20 cells (Figure 4B, 4C). The ERK inhibitor PD98059 significantly suppressed AKR1B10-mediated the migration and invasion of MCF-7 cells and BT-20 cells (Figure 5).

## DISCUSSION

In breast cancer, AKR1B10 is upregulated in the metastatic (78.0%) and recurrent (87.5%) tumors in breast cancer patients in USA, indicating its potential role in breast cancer metastasis and recurrence [2]. However, the molecular mechanisms of the action of AKR1B10 remains unclear. This study demonstrated that AKR1B10 promotes breast cancer cell invasion and migration though activation of the ERK signaling pathway. The discovery defines the role of AKR1B10 in the growth and progression of breast cancer.

In our study, we demonstrated that ectopic expression of AKR1B10 promoted breast cancer cell migration and invasion through activation of ERK signaling pathway and up-regulation of expression of MMP2 and vimentin. The ERK pathway mediates tumor metastasis via regulating the expression and activity of matrix metalloproteinases (MMPs). And it plays a critical role in ECM degradation, an important step during tumor metastasis [12–14]. As shown in Figure 4B, PD98059 (a specific inhibitor of the activation of MEK) appeared to inhibit the activation of ERK and the expression of MMP2 and vimentin in MCF-7/AKR1B10 and BT-20 cells.

We detected the AKR1B10 expression in breast cancer patients in China, and also analyzed the internal relation between AKR1B10 expression and the clinic pathological parameters of breast cancer. This result is consistent with previous research: AKR1B10 expression was correlated positively with tumor size and lymph node metastasis, but not correlate with the patient age or distant metastasis [2]. In our study, we demonstrated that ectopic expression of AKR1B10 promoted breast cancer cell migration and invasion through activation of ERK signaling pathway and up-regulation of expression of MMP2, and vimentin. The ERK signaling pathway are associated with tumor cell proliferation, invasion and migration. What interest us is that the AKR1B10 level was positively correlated with p-ERK1/2 expression. The expressions of MMP2, Vimentin protein were up-regulated in MCF-7/AKR1B10 cells compared with MCF-7/vector cell. So AKR1B10 not only is overexpressed in breast cancer, but also promotes the migration and invasion of MCF-7 cells by up-regulation AKR1B10 expression via the ERK signaling way in MCF-7 cells. The same results were observed in BT-20 cells by downregulating AKR1B10 expression with siRNA interfering. Migration refers to the behavior of the tumor cells to go to another place through deformation or movement, and vimentin promotes the deformation and movement of tumor cells. In this study, vimentin was upregulated by AKR1B10 and induced the deformation and movement of MCF-7/AKR1B10 and BT-20 cells. Invasion refers to the behavior of tumor cells to break through the basement membrane and infiltrate surrounding tissues; and the process of invasion also includes degradation of the basement membrane and extracellular matrix. MMP2 is an important extracellular matrix kinases. In this study, MMP2 was upregulated by AKR1B10 and promoted the invasion of MCF-7/AKR1B10 and BT-20 cells by degradation of extracellular matrigel in transwell invasion assay.

Our study is consistence with recent studies. Virtakoivu et al. explored that the vimentin and ERK are upregulated in breast cancer cells, and vimentin promotes ERK activation by preventing EKR de-phosphorylation and ERK upregulates vimentin expression. The vimentin-ERK axis promotes EMT of cell metastasis of breast cancer cells by regulating Slug phosphorylation [15].

## MATERIALS AND METHODS

### Samples and clinical data

Paraffin-embedded breast cancer tissue specimens with clinical data (n=131) were obtained from the First People's Hospital of Chenzhou City, China between 2014 and 2015. All cases were classified according to the World Health Organization (WHO) classification system and staged according to the TNM system. The Patients didn't receive radiotherapy or chemotherapy prior to surgery. Normal tissue specimens (n = 70) from patients without malignant tumors were also obtained by endoscopy as controls.

### Immunohistochemistry

Paraffin-embedded tissue sections were deparaffinized in xylene, washed with ethanol, rehydrated, and then incubated with fresh 3% (v/v) H_2_O_2_ for 10 min for eliminating endogenous peroxidase activity. After rinsed with distilled water, sections were washed three times with phosphate-buffered saline (PBS) for 5 min each time, and then incubated with 10% (v/v) normal goat serum (diluted with PBS) at room temperature for 20 min. The sections were incubated with rabbit anti-AKR1B10 antibody at 37°C for 1.5h, followed by incubating with HRP-labeled secondary antibody at 37°C for 30 min. Subsequently, the sections were stained with 3, 3-diaminobenzidine (DAB) for 5-10 min and counterstained with hematoxylin.

### Stable cell line establishment

AKR1B10 cDNA was cloned into pSIN-EF2 vector, a modified lentivirus vector with puromycin resistant gene. The lentivirus vector pSIN-EF2-AKR1B10-Puromycin or control vector pSIN-EF2-Puromycin was transfected into 293T cells together with auxiliary plasmids pSPAX2 and pMD2.G to package lentivirus. After infected by the lentivirus, MCF-7/AKR1B10 monoclonal cell and MCF-7/vector cells were screened out through adding puromycin into the mediums at the concentration of 1μg/mL. The expression of AKR1B10 was detected by western blot. The MCF/AKR1B10 and MCF-7/vector cells were used in the following experiments. AKR1B10 was silenced in BT-20 cells using siRNA.

### Cell wound healing assay

1 × 10^5^ cells each well were seeded into a 6-well plate and cultured in a complete medium. When the cells grew to 75% confluence, the cell layers were wounded by a sterile pipette tip, and then washed with PBS for several times to remove cell debris, and continue to incubate with serum-free medium for 48 h. The cells migrated into wound surface, which was considered as the process of *in vitro* healing. The wound healing *in vitro* was photographed by an inverted fluorescence microscope and assessed with the rate of closure. The rate of wound healing = [(the wound width of 0 h – 48 h)/0 h wound width] × 100%.

### Transwell migration and invasion experiments

Transwell chamber: 24-well, 8.0-μm pore membranes (Corning USA) was used according to the manufacturer's protocol. 1 × 10^5^ cells per well were seeded in the upper chamber in 100μL of serum-free medium, and 600μL of complete medium was added to the lower chamber as a chemoattractant at the same time. After incubated for 24 h at 37°C, the cells remaining at the upper surface of the membrane were removed with cotton swabs, and the cells on the lower surface of the membrane are the migrated cells. After fixed with 4% paraformaldehyde and stained with 0.1% crystal violet solution, the cells that passed through the filter were photographed by inverted fluorescence microscope.

The transwell invasion assay was carried out as described above, except that 100μL of 1:8 DMEM-diluted Matrigel (BD, USA) was added to each well at 37°C for 6 h before the cells were seeded onto the membrane, followed by incubating for 48h.

### Western blot analysis

Total proteins were extracted from tissues and cells with RIPA buffer (Beyotime, China). Protein concentrations of whole extracts were measured using a BCA protein assay kit (Beyotime, China). Approximately 40μg protein extract each sample was separated using a SDS–polyacrylamide gel and transferred onto the PVDF membrane (Millipore, USA). The membranes were blocked with 5% skimmed milk and incubated with rabbit anti-AKR1B10 (1:500, self-prepared), anti-ERK1/2 (1:1000, abcam, USA), anti-pERK1/2 (1:500, Cell Signaling, USA), anti-MMP2 (1:500, abcam, USA), anti-Vimentin (1:1000, abcam, USA), and mouse antibody against β-actin (1:1000, Sigma, USA) overnight at 4°C, followed by incubation for 1 h with horseradish peroxidase (HRP)-conjugated secondary antibodies (1:10,000). After extensive washing in PBST, the expression levels of the proteins were detected by Quantity-one software (Bio-Rad Laboratories, USA) using the ECL-chemiluminescent kit.

### Statistical analysis

All experiments were performed in triplicate and statistical analysis was performed using two-sided student's t-test. For the clinical samples, AKR1B10 expression associated with the clinical parameters was analyzed with the χ^2^ test.

## CONCLUSIONS

This study demonstrated that AKR1B10 promotes breast cancer metastasis at the cell levels *in vitro* and *ex vivo* clinical data. This study also demonstrated that AKR1B10 promotes breast cancer cell invasion and migration via the ERK axis pathway. Our results show that AKR1B10, as a new critical factor in breast cancer invasion and migration, may be a potential target for metastatic intervention. Further studies on the gene regulation of AKR1B10 will reveal an internal mechanism about its role on the development and progression of cancers.

## Abbreviations

AKR1B10: Aldo–keto reductase B10
AKRs: aldehyde ketone reductase
MMP-2: matrix metalloproteinase 2
qRT-PCR: quantitative reverse transcription polymerase chain reaction.
